# Seneca Valley virus 3C protease cleaves HDAC4 to antagonize type I interferon signaling

**DOI:** 10.1128/jvi.02176-24

**Published:** 2025-02-10

**Authors:** Zijian Li, Jingjing Yang, Ruiyi Ma, Shijie Xie, Dan Wang, Rong Quan, Xuexia Wen, Jue Liu, Jiangwei Song

**Affiliations:** 1 Beijing Key Laboratory for Prevention and Control of Infectious Diseases in Livestock and Poultry, Institute of Animal Husbandry and Veterinary Medicine, Beijing Academy of Agriculture and Forestry Sciences 656308 , Beijing, China; 2 Department of Preventive Veterinary Medicine, College of Animal Science and Veterinary Medicine, Shenyang Agricultural University 98428 , Shenyang, China; 3 Department of Preventive Veterinary Medicine, College of Veterinary Medicine, Yangzhou University 614704 , Yangzhou, Jiangsu, China; University of Michigan Medical School, Ann Arbor, Michigan, USA

**Keywords:** Seneca Valley virus (SVV), HDAC4, 3C protease, cleavage, type I interferon (IFN-I)

## Abstract

Seneca Valley virus (SVV) is a newly identified pathogen that poses a notable threat to the global pig industry. SVV has evolved multiple strategies to evade host antiviral innate immune responses. However, the underlying molecular mechanisms have not yet been fully elucidated. Histone deacetylases (HDACs) have been shown to function as host antiviral innate immune factors. In this study, we examined the mechanisms underlying SVV evasion of host innate immunity and found that SVV infection induced degradation and cleavage of HDAC4. Ectopic expression of HDAC4 suppressed SVV replication, whereas siRNA-mediated knockdown of HDAC4 enhanced SVV replication. Further studies showed that the viral 3C protease (3C^pro^) degraded HDAC4 in a protease activity- and caspase pathway-dependent manner. In addition, 3C^pro^ cleaved HDAC4 at Q599, which blocked its ability to limit viral replication. We also found that HDAC4 interacted with the SVV viral RNA-dependent RNA polymerase 3D and induced its proteasomal degradation. The cleaved HDAC4 products did not block SVV replication or induce 3D degradation and did not induce type I interferon (IFN) activation and expression of IFN-stimulated genes (ISGs). Collectively, these findings identified HDAC4 as an antiviral factor with effects against SVV infection and provided mechanistic insights into how SVV 3C^pro^ antagonizes its function, which has implications for viral evasion of innate immunity.

**IMPORTANCE:**

Seneca Valley virus (SVV) is an emerging pathogen that causes vesicular disease in pigs and poses a threat to the pork industry. Histone deacetylases (HDACs) are important in the regulation of innate immunity. However, little is known about their roles in SVV infection. Our results revealed HDAC4 as an anti-SVV infection factor that targets the viral RNA-dependent RNA polymerase, 3D, for degradation. The SVV proteinase 3Cpro targets HDAC4 for degradation and cleavage, and cleavage of HDAC4 abrogated its antiviral effect. HDAC4 promotes type I interferon (IFN) signaling, and SVV 3Cpro-mediated cleavage of HDAC4 antagonized induction of type I IFN and interferon-stimulated genes (ISGs). Our findings reveal a novel molecular mechanism by which SVV 3Cpro counteracts type I IFN signaling by targeting HDAC4.

## INTRODUCTION

Seneca Valley virus (SVV), also known as Senecavirus A (SVA), belongs to the genus *Senecavirus* of the family *Picornaviridae*. SVV infection is characterized by the appearance of blisters and/or ulcerative wounds on the mouth and nose, oral mucosa, coronary bands, and hooves (1). These manifestations may progress to severe porcine blister disease and lead to neonatal death (2). As SVV infection is a new infectious disease, it causes unavoidable economic losses to the global pig breeding industry. Furthermore, the clinical differentiation of SVV from vesicular stomatitis virus of swine (VESV), vesicular stomatitis virus (VSV), swine vesicular disease virus (SVDV), and foot-and-mouth disease virus (FMDV) is challenging (3).

SVV has the standard genome structure of a small ribonucleic acid virus of type L-4-3-4 (L-VP4-VP2-VP3-VP1-2A-2B-2C-3A-3B-3C-3D). Its genome is 7.2 kb in length (4) and encodes a single polypeptide that is processed by proteases 2A and 3C to generate structural and non-structural proteins. This polypeptide, which is comprised of a leading protein and three major protein domains (P1, P2, and P3) (5), is processed into mature viral proteins by 3C^pro^, a virus-encoded cysteine protease that contains a conserved His-Asp-Cys catalytic triad (6). SVV has developed several strategies to evade the host’s innate immune responses. For example, 3C^pro^ dampens type I interferon (IFN) activity by degrading and cleaving numerous crucial proteins in the innate immune pathway (7–10). Specifically, to escape host immunity SVV 3C^pro^ targets key innate immune adapters for cleavage, such as TIR-domain-containing adapter-inducing IFN-β (TRIF), mitochondrial antiviral-signaling protein (MAVS), TRAF family member-associated NF-κB activator (TANK) (7), cyclic GMP-AMP synthase (cGAS) (8), transducer and activator of transcription 2 (STAT2) (10), and optineurin (OPTN) (11). Moreover, SVV can block the phosphorylation of interferon regulatory factor 3 (IRF3) and subsequently decrease the expression of IFN-β and interferon-stimulated gene 56 (ISG56) by degrading retinoic acid-inducible gene-I (RIG-I) (12). SVV 3C^pro^-mediated suppression of RIG-I and TANK-binding kinase 1 (TBK1)-induced type I IFN production is dependent on its deubiquitinating (DUB) activity (9).

Histone deacetylases (HDACs) are a group of enzymes that participate in innate immune cell responses and are key targets in inflammatory diseases (13, 14). Human HDACs are classified into five subfamilies: I, IIa, IIb, III, and IV (15), which include the following members: class I (HDAC1, HDAC2, HDAC3, and HDAC8), class IIa (HDAC4, HDAC5, HDAC7, and HDAC9), class IIb (HDAC6 and HDAC10), class III (Sirt1 to Sirt7), and class IV (HDAC11) (16). HDACs deacetylate histones, which facilitates the formation of a tight histone-DNA complex, resulting in repression of gene replication and transcription (17). They also govern the replication and transcription of various genes encoding innate immune mediators and regulate innate immune responses to viral infections (18–24).

HDAC4, a class II HDAC, is devoid of regularly arranged non-polar residues and an extensive hydrophobic core. The glutamine-rich domain, which contains 19 glutamines within 68 residues, folds into a straight α-helix and forms a tetramer. Generally, these glutamine-rich motifs play a role in mediating protein-protein interactions (25). HDAC4 has been implicated in the modulation of hepatitis B virus (HBV) replication (26) and promoted type I interferon signaling, restricted DNA viruses, and was degraded by vaccinia virus protein C6 (24). In the absence of HDAC4 expression, several influenza A virus (IAV)-induced activities were downregulated, including phosphorylation of STAT1 and the expression of ISGs, such as interferon-induced transmembrane protein 3 (IFITM3), ISG15, and viperin (18). HDAC5 promoted TRIF-mediated IRF3 activation and restricted the replication of multiple orthopoxviruses, and the C6 protein of these viruses induced the degradation of HDAC5 (19). Porcine deltacoronavirus (PDCoV) nonstructural protein 5 (nsp5), a 3C-like protease, cleaved HDAC2, and the cleaved products did not inhibit PDCoV replication (20). HDAC6 interacted with PDCoV nsp8, resulting in its deacetylation activity-dependent proteasomal degradation (21). Porcine epidemic diarrhea virus (PEDV) infection led to reduced expression of HDAC1 and inhibition of HDAC1-impaired type I IFN signaling pathways and ISG expression (22). These studies showed that HDACs increase the resistance of cells to viral infection. However, whether HDACs participate in the regulation of SVV replication remains unknown.

In the present study, we identified HDAC4 as an antiviral protein against SVV. Mechanistically, we showed that HDAC4 interacts with SVV 3D protein, resulting in proteasomal degradation. SVV infection downregulates HDAC4 expression, and SVV 3C^pro^ cleaves and degrades HDAC4 to antagonize its antiviral effects. Furthermore, the cleaved HDAC4 products dampen activation of the type I IFN pathway and subsequent transcription of ISGs. Thus, we revealed a novel mechanism by which SVV escapes the host antiviral immune response.

## MATERIALS AND METHODS

### Cells, viruses, antibodies, and reagents

The BHK-21 (baby hamster kidney-21) cells, HEK-293T (human embryonic kidney 293T) cells, and PK-15 (porcine kidney-15) cells were cultured and maintained within Dulbecco’s modified Eagle’s medium (DMEM, Gibco) supplement with 10% fetal bovine serum (FBS). As previously described, SVV strain CHhb17 (MG983756.1) and SVV VP1 mouse monoclonal antibody utilized in this research were as described previously (27). The rescued Seneca Valley virus with eGFP inserted (rSVV-eGFP) was generously provided by Dr. Fuxiao Liu (Qingdao Agricultural University) (28). The GFP rabbit polyclonal antibody (50430-2-AP), Flag rabbit polyclonal antibody (20543-1-AP), Flag mouse monoclonal antibody (66008-4-Ig), and β-actin mouse monoclonal antibody (66009-1-Ig) were acquired from Proteintech (Wuhan, China). The rabbit anti-HA antibody (3724) was purchased from Cell Signaling Technology (Beverly, MA, USA). Lambin B1 (ab16048) was obtained from Abcam (Cambridge, MA, USA). Alexa-568-labeled goat anti-rabbit fluorescence secondary antibody (11011) and Alexa-488-labeled goat anti-mouse fluorescence secondary antibody (11011) were obtained from Invitrogen (CA, USA). IFN-alpha/beta (HY-P72613) was purchased from MedChemExpress (MCE, Shanghai, China). Bafilomycin A1 (Baf A1, S1413), chloroquine (CQ, S6999), 3-methyladenine (3-MA, S2767), MG132 (S2619), Z-VAD-FMK (S7023), diABZI (S8796), and Lopinavir (S1380) were obtained from Selleck Chemicals (Shanghai, China).

### Plasmid construction

The GFP-tagged expression plasmids encoding SVV proteins, as well as GFP-3C and its mutation (H48A, C160A, double mutation H48A, and C160A), have been employed in our previous studies (10, 11). The HDAC4 gene (NM_006037.3) was synthesized from RuiBiotech Biotechnology Co., Ltd. (Beijing, China) and reconstructed into the p3 × FLAG-CMV-10 vector (E4401, Sigma) using a one-step DNA assembly kit (D0204P, Lablead, Beijing, China). Flag-tagged HDAC4 truncations were produced based on the HDAC4 backbone. The site-mutated HDAC4 was produced by site-directed mutagenesis with KOD DNA polymerase (KFX-201, TOYOBO, Japan).

### Western blotting

The cell lysate was centrifuged at 13,000 × *g* for 10 min at 4°C. The supernatant was retrieved and boiled in 5 × loading buffer for 10 min. Samples were separated by sodium dodecyl sulfate-polyacrylamide gel electrophoresis (SDS-PAGE). The gel-containing proteins were transferred to a nitrocellulose membrane (66485, PALL, USA). The membrane was blocked with 5% skim milk for 1 hour, incubated overnight at 4°C with specified primary antibodies, and then for 1 hour with horseradish peroxidase (HRP)-conjugated anti-rabbit, or anti-mouse secondary antibodies at room temperature (RT), respectively. Proteins were visualized using an enhanced ECL chemiluminescence substrate kit (H1060, Lablead, Beijing, China).

### Co-immunoprecipitation (Co-IP)

HEK-293T cells or BHK-21 cells were transfected with indicated plasmids. After 24 h post-transfection, the cells were harvested and lysed with lysis buffer (AIWB-012, Affibody, Wuhan, China). The cell lysate was centrifuged at 13,000 × *g* for 10 min at 4°C. The lysate was incubated with anti-GFP magnetic beads (P2132, Beyotime, Shanghai, China) or anti-Flag magnetic beads (HY-K0207, MCE, Shanghai, China) overnight at 4°C. The beads were washed three times with lysis buffer. The immunoprecipitates were boiled with 5 × loading buffer for 10 min. Subsequently, the immunoprecipitates were subjected to western blotting.

### Quantitative RT-PCR

Total RNA was extracted from the cells using the FastPure Cell/Tissue Total RNA Isolation Kit-BOX 2 (RC101-01, Vazyme, Nanjing, China). 1,000 ng RNA was employed to synthesize cDNA with the first-strand synthesis master mix (F0202, Lablead, Beijing, China). The mRNAs were quantified via one-step real-time RT-PCR using EasyQ SYBR qPCR mix (TSQ0102, Yeasen, Shanghai, China), and quantitative real-time PCR was repeated using the Bio-Rad CF96 real-time PCR system. Data were normalized to the glyceraldehyde-3-phosphate dehydrogenase (GAPDH) level of each sample, the relative gene expression by calculating 2^−ΔΔCT^ (where CT represents the threshold cycle). The primers are as follows: for GAPDH, forward primer 5′-GCCATCAATGACCCCTTCATTGA-3′ and reverse primer 5′-ATCTCGCTCCTGGAAGATGGTGAT-3′; for HDAC4, forward primer 5′-ATGGACTTTCTGGCCGAGACCA-3′ and reverse primer 5′-AACTGGTGGTCCAGGCGCAGGT-3′. The primer sequences are listed in Table 1.

**TABLE 1:** Primers used in this study

Primer^*a*^	Sequence (5′-3′)
Human IFN-β-F	TCTTTCCATGAGCTACAACTTGCT
Human IFN-β-R	GCAGTATTCAAGCCTCCCATTC
Human ISG60-F	AGCTACAATGTACAACTTGT
Human ISG60-R	CATCTGAGAGTCTGCCCAAG
Human ISG56-F	CCTCCTTGGGTTCGTCTACA
Human ISG56-R	GGCTGATATCTGGGTGCCTA
Human OAS1-F	GACGATGAGACCGACGATCC
Human OAS1-R	GTGCAGGTCCAGTCCTCTTC
Human Mx1-F	CAGGACATTTGAGACAATCGTG
Human Mx1-R	TCGAAACATCTGTGAAAGCAAG

^*a*^ F denotes forward PCR primer; R denotes reverse PCR primer.

### Indirect immunofluorescence (IFA)

Cells were fixed with 4% paraformaldehyde for 10 min, then permeabilized with 0.1% Triton X-100 in 2% bovine serum albumin (BSA) for 15 min. After blocking non-specific binding by incubating the cells with 2% BSA for 30 min (RT), the cells were incubated with the suitable primary antibody overnight at 4°C. After washed three times with phosphate-buffered saline (PBS), Alexa Fluor 561/488-conjugated secondary antibodies (from Invitrogen) were incubated for 1 h (RT). Subsequently, the cells were washed three times with PBS and stained with DAPI. Sample images were captured using a Nikon Al confocal microscope (Tokyo, Japan).

### RNA interference (RNAi)

The small interfering RNAs (siRNAs) targeting HDAC4 were manufactured by GenePharma. (Suzhou, China). The siRNA target sequences were as follows: si-HDAC4-1 (sense, 5′-CAGCUUCUGAACCGAAUCUTT-3′; antisense, 5′-AGAUUCGGUUCAGAAGCUGTT-3′); si-HDAC4-2 (sense, 5′-GCAGGAUACUGAGAGACUUTT-3′; antisense, 5′-AAGUCUCUCAGUAUCCUGCTT-3′); siNC (sense, 5′-UUCUCCGAACGUGUCACGUTT-3′; antisense, 5′-ACGUGACACGUUCGGAGAATT-3′), which was used as a negative control. Both human and Mesocricetus auratus HDAC4 were the targets of si-HDAC4-1, while Mesocricetus auratus HDAC4 was the target of si-HDAC4-2. The siRNAs at a final concentration of 20 pmol were transfected into cells using RNAiMAX transfection reagent (13778150, Thermo Fisher).

### TCID_50_ assay

The BHK-21 cells in six-well plates were infected at the multiplicity of infection (MOI) indicated. After 1 h of incubation at 37°C, cells were washed three times with DMEM. Then the cells were overlaid with DMEM containing 2% FBS. At the indicated times post-infection, cells were harvested. After three freeze-thaw cycles, samples were titrated on BHK-21 cells using TCID_50_ assay.

### Nuclear and cytoplasmic fractionation

BHK-21 cells infected with SVV or transfected with plasmids were harvested at indicated times, and nuclear and cytoplasmic fractions were isolated using the nuclear and cytoplasmic extraction kit (Thermo Fisher, 78833) according to the manufacturer’s instructions. The nuclear and cytoplasmic components were subjected to western blotting with antibodies against β-actin and Lamin B1, respectively.

### Luciferase reporter assay

HEK-293T cells cultured in 24-well plates were co-transfected with either an empty vector as a control or the indicated plasmids, along with the pRL-TK plasmid and the pISRE-Luc plasmid. Twenty-four hours after transfection, IFN-α was employed to treat the cell samples. Subsequently, the cells were lysed and analyzed through luciferase activity assays with the dual luciferase reporter assay kit (DL101-01, Vazyme, Nanjing, China) in accordance with the manufacturer’s guidelines using Renilla luciferase as a reference.

### Statistical analysis

Data are presented as the means ± standard deviations (SDs) and were analyzed using GraphPad Prism software. *P* values of < 0.05 were considered statistically significant.

## RESULTS

### SVV infection results in the cleavage and degradation of HDAC4

**Fig 1:**
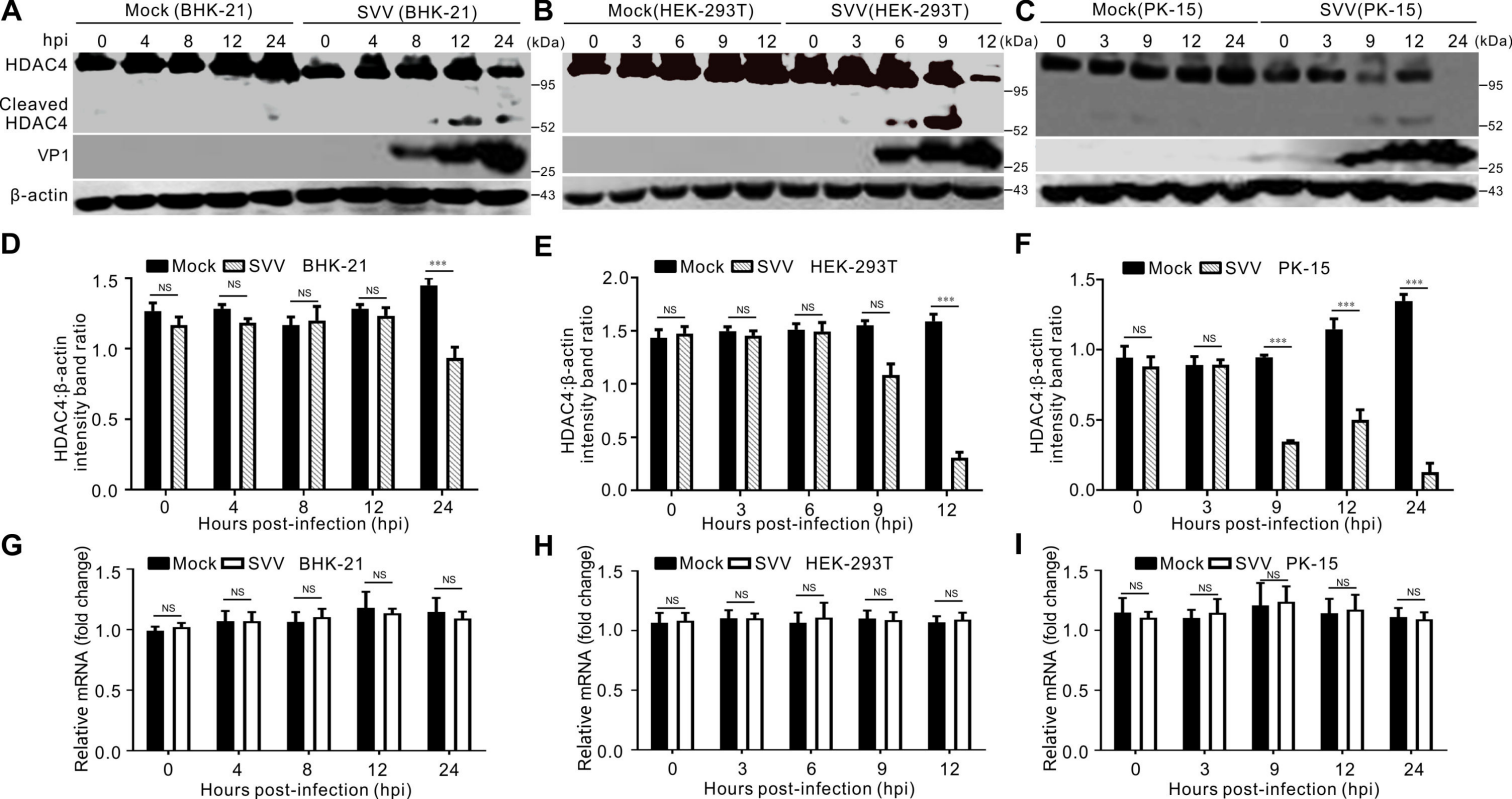
SVV infection induces HDAC4 cleavage and degradation. (**A–C**) Western blot analysis of HDAC4 protein levels in BHK-21 cells (**A**), HEK-293T cells (**B**), and PK-15 cells (**C**) in six-well plates infected with SVV at an MOI of 5 or mock infected with PBS, respectively. SVV VP1 capsid protein was served as an infection indicator and β-actin used as an internal control. (**D–F**) The graph shows quantification analysis levels of HDAC4 normalized against β-actin from figure (**A–C**) with ImageJ. Data are shown as the mean ± standard deviation (SD) obtained from three independent experiments. (**G–I**) Relative abundance of HDAC4 mRNA in SVV-infected BHK-21 cells (**G**), HEK-293T cells (**H**), and PK-15 cells (**I**) at different time points post-infection were determined by qRT-PCR. The level of HDAC4 mRNA was normalized against GAPDH. Data are shown as the mean ± standard deviation (SD) obtained from three independent experiments. (***P＜0.001; NS, not significant).

Host HDACs have been reported to regulate the replication of various viruses (18–24). Conversely, viruses attenuate cellular deacetylase activity to facilitate viral replication. Therefore, we examined HDAC4 expression in baby hamster kidney-21 (BHK-21), human embryonic kidney 293T (HEK-293T), and porcine kidney-15 (PK-15) cells infected with SVV strain CHhb17. The results showed that after SVV infection, HDAC4 was degraded and cleaved, generating a band that was detectable with a specific HDAC4 antibody, whereas in mock-infected cells, HDAC4 remained unchanged (Fig. 1A through F). However, the levels of HDAC4 mRNA were not significantly decreased after SVV infection (Fig. 1G through I). This suggests that the decrease in HDAC4 expression is not attributable to a reduction in mRNA levels. These results indicate that SVV infection induces HDAC4 degradation and cleavage.

### HDAC4 is an antiviral factor against SVV infection

**Fig 2:**
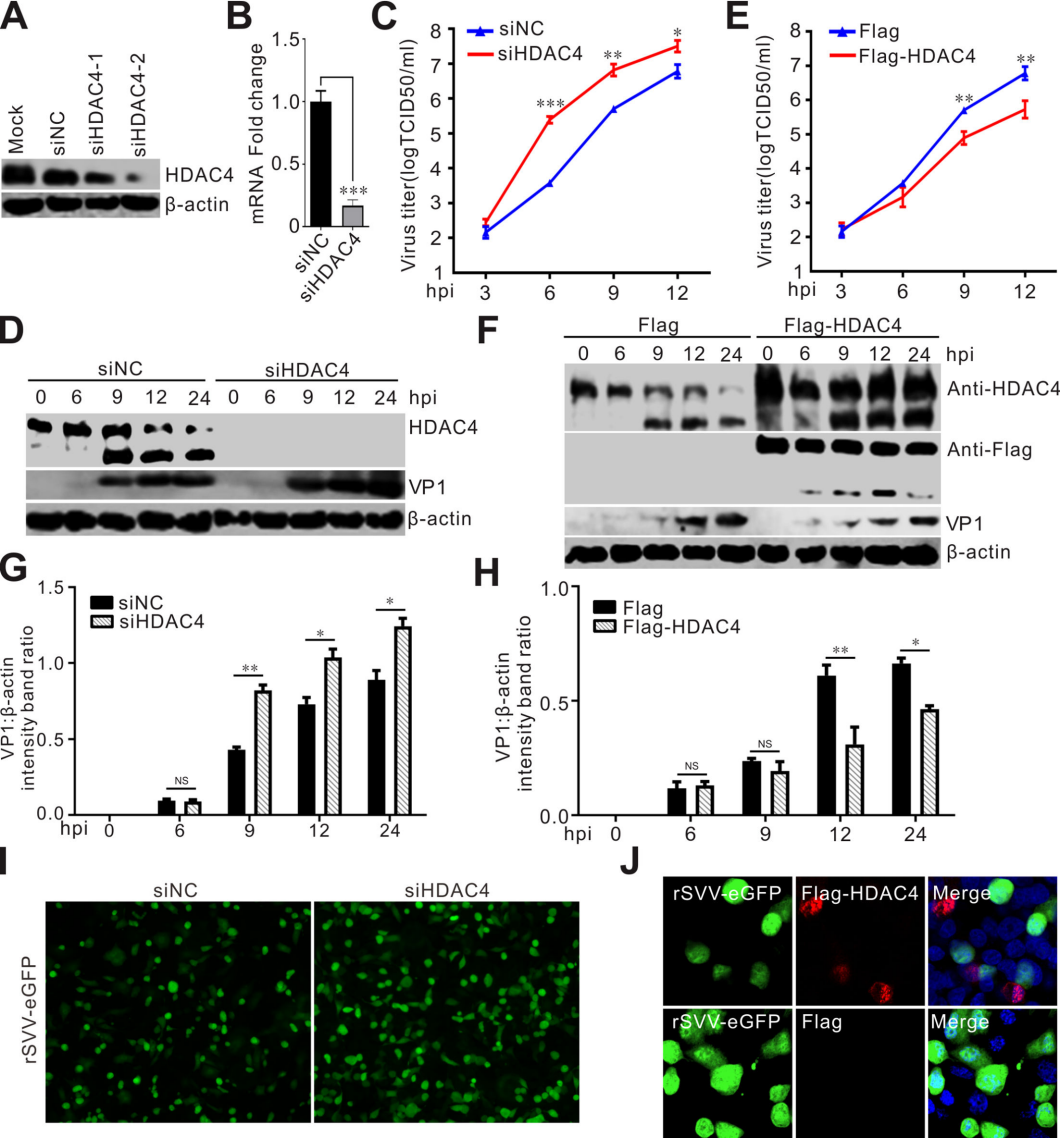
HDAC4 is an antiviral factor against SVV infection. (**A-B**) Western blot (**A**) and RT-qPCR (**B**) analysis of the silencing efficiency for HDAC4. BHK-21 cells were transfected with siRNA for HDAC4 at a concentration of 20 pmol. siNC transfected cells were used as negative controls. (**C-D**) BHK-21 cells were transfected with siHDAC4 or siNC for 24 h and then infected with SVV and harvested at indicated times. TCID_50_ (**C**) and Western blot (**D**) were exploited to analyze the replication of SVV in HDAC4-silenced BHK-21 cells. (**E-F**) BHK-21 cells were transfected with Flag-HDAC4 or Flag empty vector for 24 h and then infected with SVV and harvested at indicated times. TCID_50_ (**E**) and Western blot (**F**) were exploited to analyze the replication of SVV in HDAC4-silenced BHK-21 cells. (**G-H**) Quantification analysis of VP1 expression from (**D-F**) with Image J, respectively, and the level of VP1 band ratios was normalized to β-actin. (**I-J**) BHK-21 cells were transfected with siHDAC4 or siNC (**I**), Flag-HDAC4 or Flag empty vector (**J**) for 24 h and then infected with rSVV-eGFP, Flag-HDAC4 was stained with anti-Flag antibody (red), then captured under confocal microscopy at 12 h post-infection (hpi). The error bars indicated mean ± SD from three independent experiments (*P＜0.05; **P＜0.01; ***P＜0.001; NS, not significant).

Next, we investigated the effects of HDAC4 expression on SVV by knocking down HDAC4 using RNA interference (RNAi) with two small interfering RNAs (siRNAs) that target different regions of HDAC4. Analysis showed that siRNA-mediated knockdown markedly reduced the transcription and expression of HDAC4 (Fig. 2A and B). We also observed that viral replication was increased following SVV infection in HDAC4-depleted cells (Fig. 2C, D, and G). Conversely, overexpression of HDAC4 suppressed SVV replication (Fig. 2E, F and H). We then used a recombinant eGFP-expressing SVV (rSVV-eGFP) to infect BHK-21 cells to verify the effect of HDAC4 on SVV replication (Fig. 2I and J). Knockdown of HDAC4 enhanced the fluorescence signal from rSVV-eGFP (Fig. 2I), whereas this fluorescence signal was remarkably reduced in HDAC4-overexpressing cells (Fig. 2J). These results confirm that HDAC4 negatively affects SVV replication.

### SVV 3C^pro^ cleaves and degrades HDAC4

**Fig 3:**
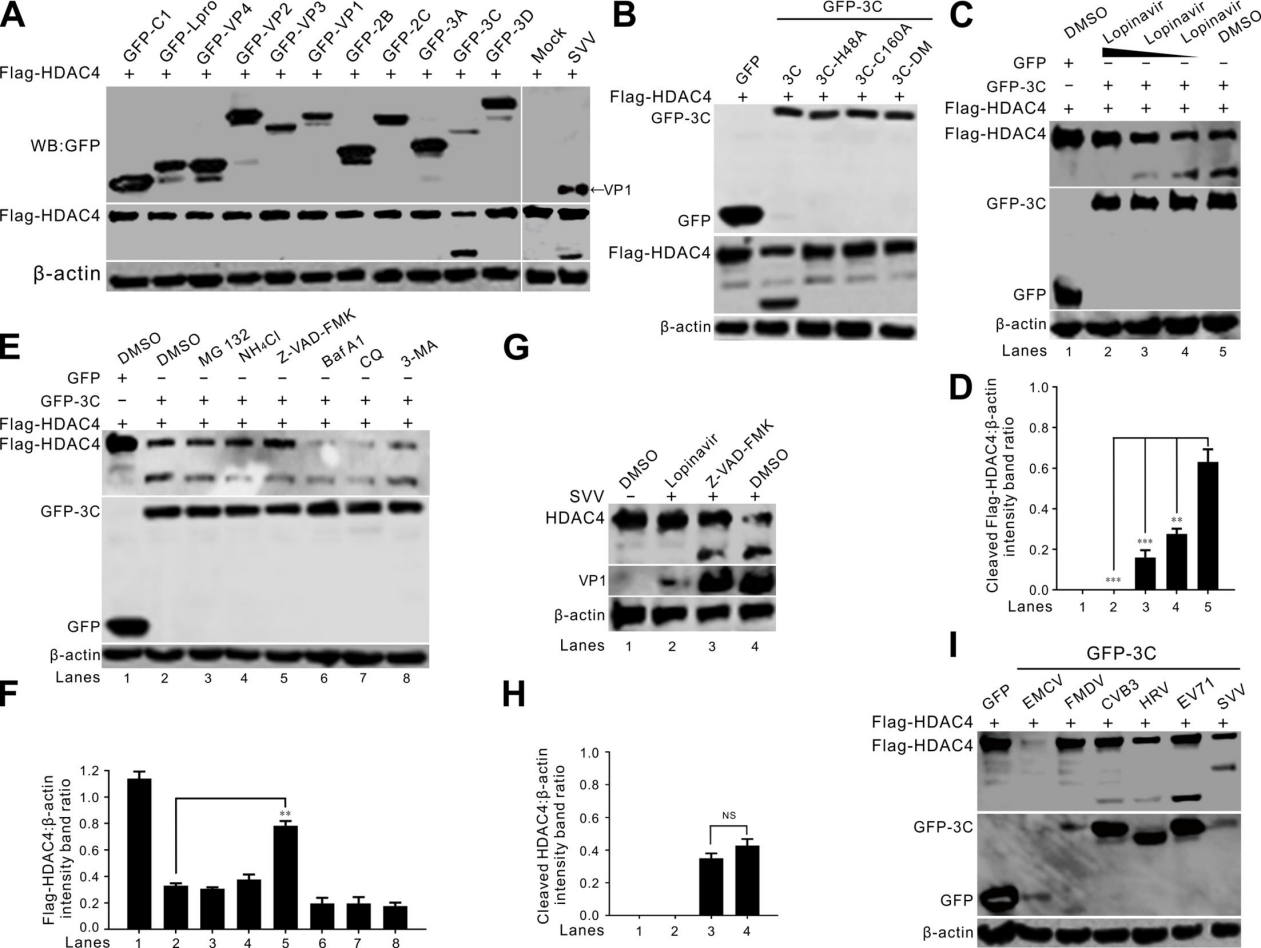
SVV 3C^pro^ cleaves and degrades HDAC4. (**A**) Western blot analysis of Flag-HDAC4 protein levels in BHK-21 cells cotransfected with plasmids expressing GFP-tagged SVV protein and SVV infection. Cell lysates were collected at 24 hours post-transfection (hpt) and 12 hpi, then subjected to immunoblotting with antibodies against GFP, Flag, VP1, and β-actin as an internal control. (**B**) Western blot analysis of Flag-HDAC4 protein levels in BHK-21 cells cotransfected with GFP-3C, GFP-3C^H48A^, GFP-3C^C160A^, GFP-3C^DM^, or GFP empty vector, respectively. (**C**) Western blot analysis of Flag-HDAC4 protein levels in BHK-21 cells cotransfected with GFP-3C or GFP empty vector for 12 h, then cells were treated with Lopinavir (40 µM, 20 µM, and 5 µM) for 12 h, respectively. (**D**) Quantification analysis of protein expression from (**C**) with Image J, the level of cleaved Flag-HDAC4 band ratios was normalized to β-actin. (**E**) Western blot analysis of Flag-HDAC4 protein levels in BHK-21 cells cotransfected with GFP-3C or GFP empty vector for 24 h, then cells were treated with MG132 (10 µM), NH_4_Cl (10 mM), Z-VAD-FMK (50 µM), Baf A1 (200 nM), CQ (40 µM), and 3-MA (25 mM) for 12 h, respectively. (**F**) Quantification analysis of protein expression from (**E**) with Image J, the level of full-length Flag-HDAC4 band ratios was normalized to β-actin. (**G**) Western blot analysis of HDAC4 protein levels in BHK-21 cells infected with SVV for 12 h, treated with Lopinavir (40 µM) and Z-VAD-FMK (50 µM). (**H**) Quantification analysis of protein expression from (**G**) with Image J, the level of cleaved HDAC4 band ratios was normalized to β-actin. (**I**) Flag-HDAC4 was cotransfected with a GFP empty vector or GFP-tagged 3C^pro^ of EMCV, FMDV, CVB3, HRV, EV71, and SVV. Western blot analysis of Flag-HDAC4 protein expression levels at 24 hpt. Data are shown as the mean ± standard deviation (SD) obtained from three independent experiments. (**P＜0.01; ***P＜0.001; NS, not significant).

To determine which SVV proteins are involved in the degradation of HDAC4, we cotransfected BHK-21 cells with Flag-tagged HDAC4 and a series of SVV protein-expressing plasmids. We observed degradation and cleavage of HDAC4 only in cells expressing SVV 3C^pro^, suggesting that 3C^pro^ is solely responsible for HDAC4 degradation and cleavage (Fig. 3A). Expression of 3C^pro^ resulted in the generation of an HDAC4 cleavage fragment similar to that observed during SVV infection (Fig. 3A). We then investigated whether the protease activity of 3C^pro^ was required for HDAC4 degradation and cleavage. BHK-21 cells were cotransfected with Flag-HDAC4 and vectors expressing GFP-tagged 3C proteins containing mutations in the catalytic triad, that is, GFP-3C^H48A^, GFP-3C^C160A^, and a double mutant, GFP-3C^H48A-C160A^ (GFP-3C^DM^). The results showed that H48 and C160 of 3C^pro^ contributed to the degradation and cleavage of HDAC4, and 3C^pro^ without protease activity failed to degrade and cleave HDAC4 (Fig. 3B). Lopinavir inhibits the cleavage of HDAC4 induced by 3C^pro^ (Fig. 3C and D). Treatment with the pan-caspase inhibitor Z-VAD-FMK also blocked the degradation of HDAC4, indicating that HDAC4 degradation is caspase-dependent (Fig. 3E and F). Z-VAD-FMK exerts no suppressive effect on 3C^pro^-mediated or SVV infection-mediated HDAC4 cleavage (Fig. 3E through H). We also observed that 3C^pro^ proteins from other picornaviruses, including CVB3, HRV, and EV71, cleaved HDAC4 (Fig. 3I). However, the cleavage bands generated by SVV 3C^pro^ were larger than those generated by 3C^pro^ from CVB3, HRV, and EV71, suggesting different cleavage site specificity for the 3C^pro^ proteins from various picornaviruses (Fig. 3I). These results verify that SVV 3C^pro^ induced HDAC4 degradation and cleavage is protease activity-dependent.

### Cleavage of HDAC4 at Q599 by SVV 3C^pro^ abrogates its antiviral activity

**Fig 4:**
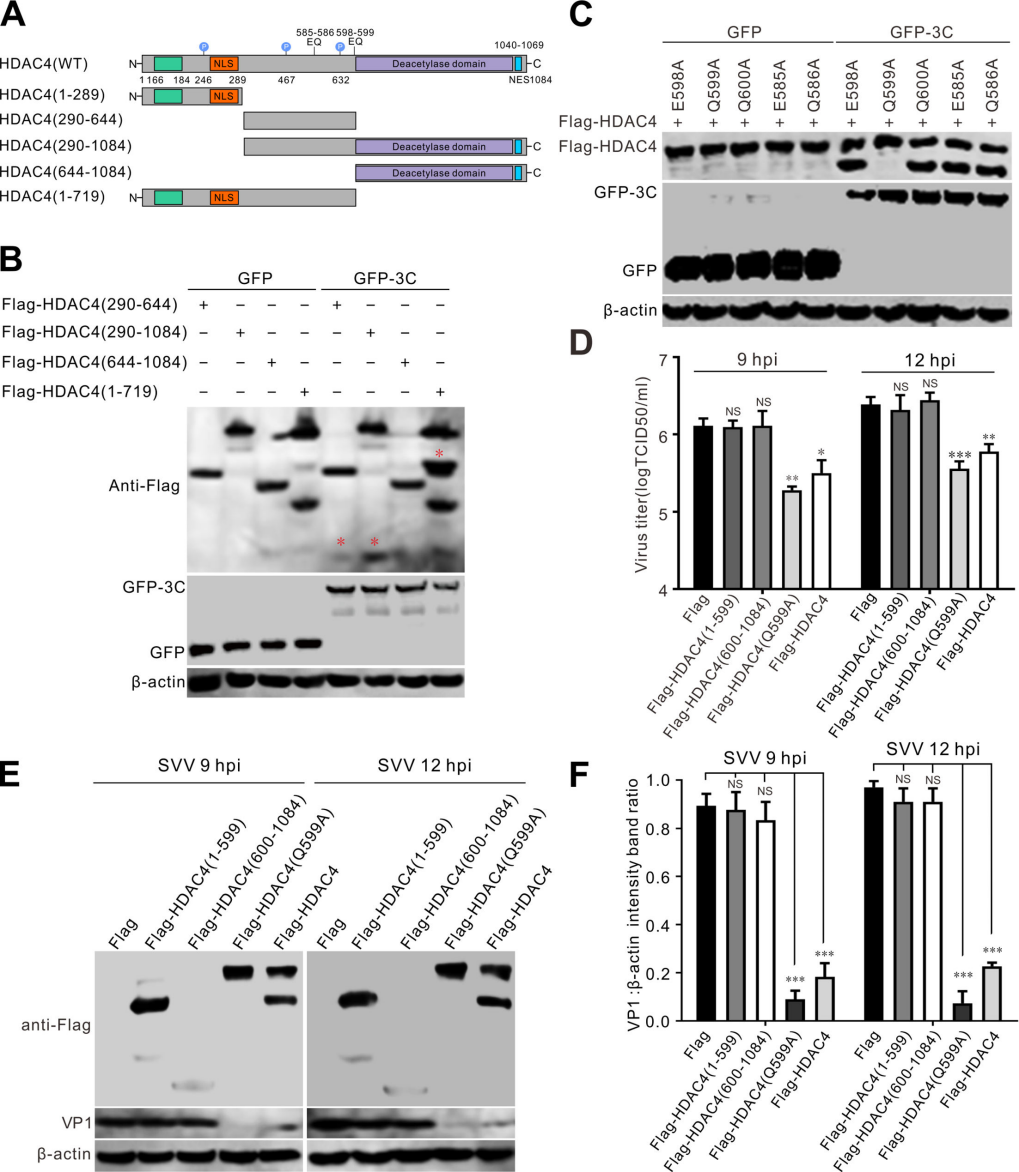
Cleavage of HDAC4 at Q599 by SVV 3C^pro^ abrogates its antiviral activity. (**A**) Domain organization of HDAC4. (**B-C**) The truncation constructs (**B**) and point mutations (**C**) of Flag-HDAC4 were cotransfected with a GFP empty vector or GFP-3C. Cell lysates were collected at 24 hpt and subjected to immunoblotting with antibodies against GFP, Flag, and β-actin as an internal control. (**D-E**) BHK-21 cells cotransfected with Flag-HDAC4 (1–599), Flag-HDAC4 (600–1,048), Flag-HDAC4 (Q599A), Flag-HDAC4, or Flag empty vector for 24 h, and then infected with SVV for 9 h and 12 h, respectively. The virus titers and VP1 production were examined by TCID_50_ assay (**D**) and western blot (**E**), respectively. Data are shown as the mean ± standard deviation (SD) obtained from three independent experiments. (**F**) The level of VP1 production from (**E**) was quantified and normalized against β-actin with Image J. Data are shown as the mean ± standard deviation (SD) obtained from three independent experiments. (*P＜0.05; **P＜0.01; ***P＜0.001; NS, not significant).

Previous studies showed that picornavirus 3C^pro^ preferentially recognizes glutamine-glycine (Q-G) or glutamic acid-glutamine (E-Q) residues as cleavage sites (29). Initially, we constructed plasmids encoding truncated HDAC4 proteins based on the structure and molecular weight of the cleaved HDAC4 fragments, including Flag-HDAC4 (1–289), Flag-HDAC4 (290–644), Flag-HDAC4 (290–1,084), Flag-HDAC4 (644–1,084), and Flag-HDAC4 (1–719) (Fig. 4A). BHK-21 cells were cotransfected with GFP-3C or GFP empty vector and one of these truncation plasmids. We observed that Flag-HDAC4 (290–644), Flag-HDAC4 (290–1,084), and Flag-HDAC4 (1–719) were cleaved by GFP-3C (Fig. 4B). This indicated that the cleavage site of HDAC4 is positioned within residues 290–719. Next, we mutated Q or E residues in this region (290–719) to alanine (A), generating Flag-HDAC4 (E585A), Flag-HDAC4 (Q586A), Flag-HDAC4 (E598A), Flag-HDAC4 (Q599A), and Flag-HDAC4 (Q600A). BHK-21 cells were cotransfected GFP-3C or an empty GFP vector with one of these point mutation plasmids. We observed that Flag-HDAC4 (Q599A) was resistant to SVV 3C^pro^-mediated cleavage, while the other mutants were not (Fig. 4C). Next we transfected BHK-21 cells with Flag-HDAC4-WT (wild type), Flag-HDAC4 (1–599), Flag-HDAC4 (600–1,084), or a Flag empty vector for 24 h, and then infected these cells with SVV for 9 and 12 h. In contrast to HDAC4-WT, the constructs carrying the two cleavage products, HDAC4 (1–599) and HDAC4 (600–1,084), were unable to suppress SVV replication (Fig. 4D through F). However, the inhibitory effect of HDAC4 (Q599A) on SVV infection was stronger than that of HDAC4-WT (Fig. 4D through F). These findings indicate that the cleavage products of HDAC4 cannot block SVV replication.

### HDAC4 interacts with viral RNA-dependent RNA polymerase 3D protein

**Fig 5:**
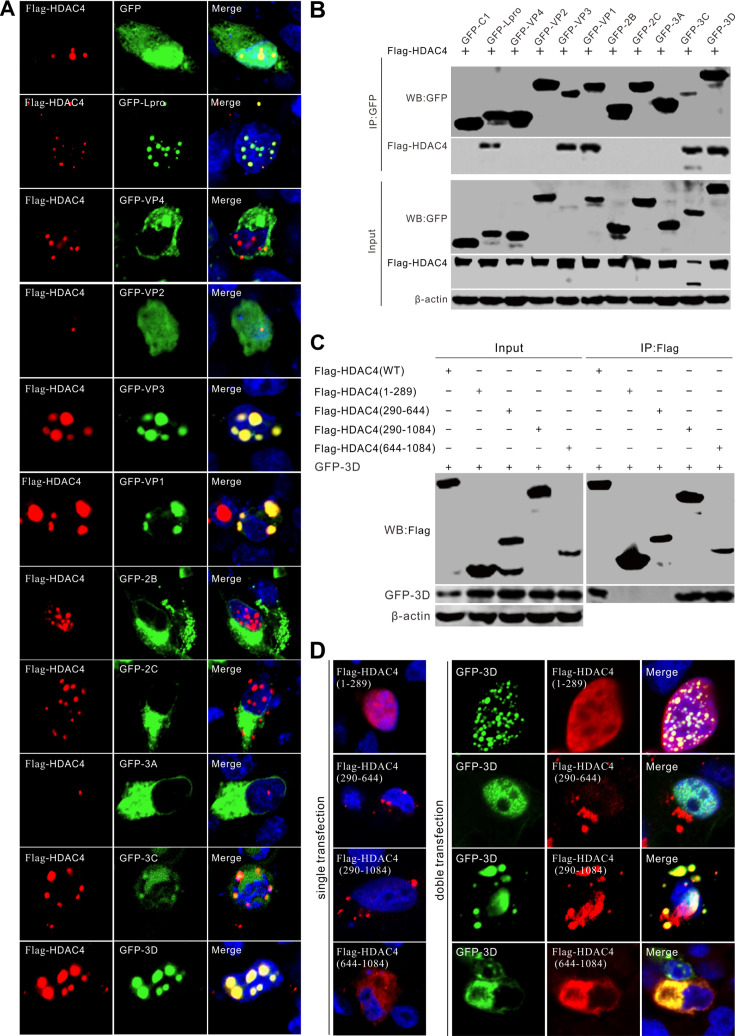
HDAC4 interacts with viral Lpro, VP3, VP1, 3C, and 3D proteins. (**A**) Colocalization analysis of Flag-HDAC4 in BHK-21 cells cotransfected with plasmids expressing GFP-tagged SVV protein. Cells were fixed at 24 hpt and stained with antibodies to Flag (red) and DAPI (blue), and examined under confocal microscopy. (**B**) Plasmids expressing GFP-tagged SVV protein were cotransfected with Flag-HDAC4 for 24 h, then subjected to co-IP analysis with antibodies against GFP, Flag, and β-actin as an internal control. (**C**) Interaction domains identification of Flag-HDAC4 with GFP-3D. Flag-HDAC4 and its truncation constructs were cotransfected with GFP-3D for 24 h, then subjected to co-IP analysis with antibodies against GFP, Flag, and β-actin as an internal control. (**D**) Colocalization analysis of Flag-HDAC4 truncation constructs with GFP-3D. BHK-21 cells were cotransfected with indicated plasmids for 24 h and stained with antibodies to Flag (red) and DAPI (blue), and examined under confocal microscopy.

Confocal microscopy and co-immunoprecipitation (Co-IP) assays were used to identify the viral proteins that interact with HDAC4. As shown in Fig. 5A, five viral proteins, Lpro, VP3, VP1, 3C, and 3D, colocalized with HDAC4 and exhibited punctate distributions (Fig. 5A). Co-IP assays confirmed these interactions (Fig. 5B). The C-terminal deacetylase domain of HDAC4 is involved in its interaction with 3D (Fig. 5C). We found that HDAC4-WT degraded the viral 3D protein, whereas the individual N- and C-terminal domains of HDAC4 lacked the ability to degrade 3D (Fig. 5C). HDAC4 has a nuclear localization signal (NLS) sequence, and ectopically expressed HDAC4 was mainly located in the nucleus (Fig. 5A). HDAC4 constructs lacking the NLS, HDAC4 (290–644), HDAC4 (290–1084), and HDAC4 (644–1084) were located in the cytoplasm (Fig. 5D). However, HDAC4 (290–1084) and HDAC4 (644–1084) still colocalized with 3D (Fig. 5D), which is in accordance with the results shown in Fig. 5C. These results suggest that the deacetylase domain of HDAC4 interacts with SVV 3D protein.

### HDAC4 targets viral 3D protein for proteasomal degradation

**Fig 6:**
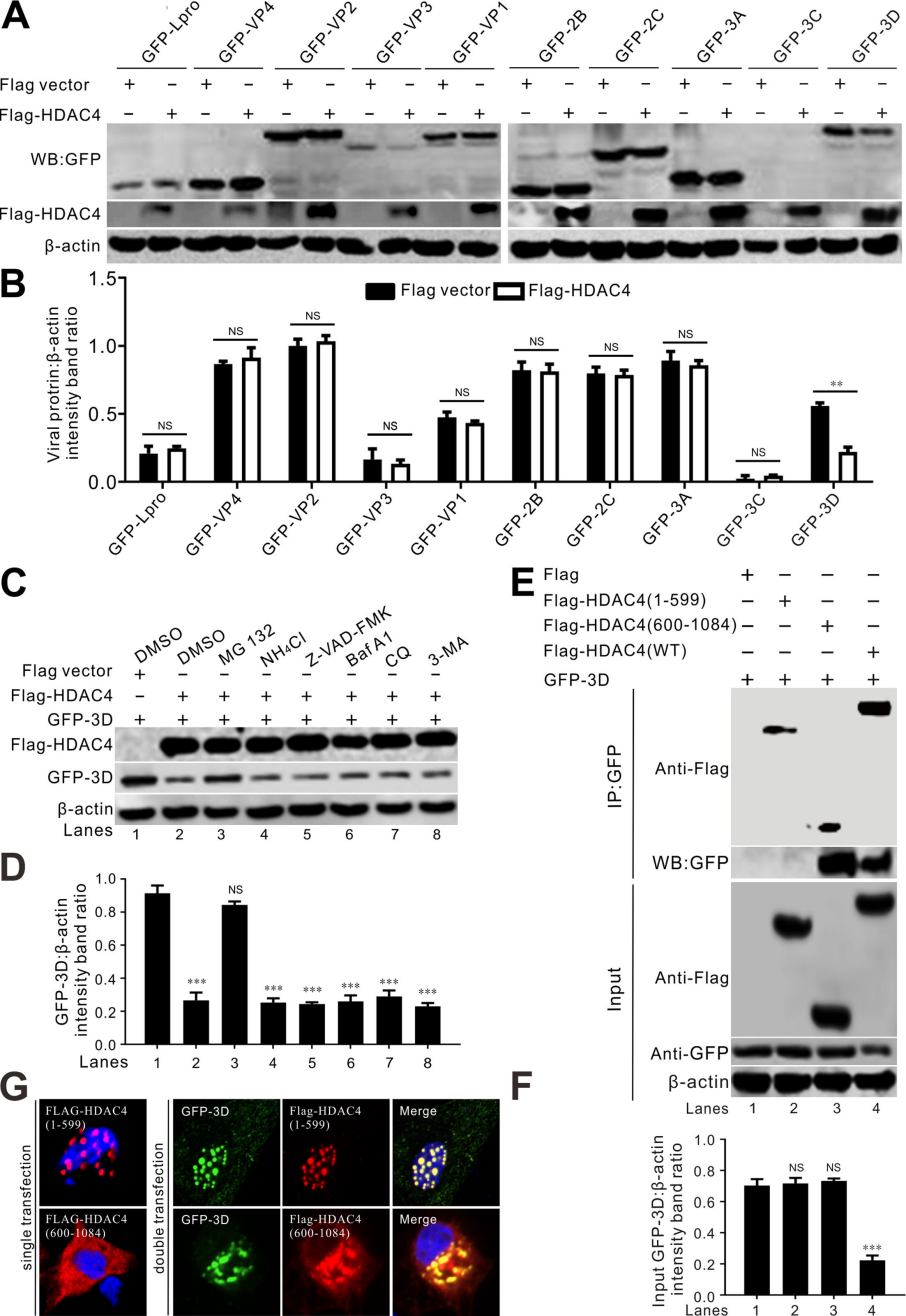
HDAC4 targets the viral 3D protein for proteasomal degradation. (**A**) Western blot analysis of viral protein levels in BHK-21 cells cotransfected with Flag-HDAC4 with individual GFP-tagged SVV protein-expressing plasmids. Cell lysates were collected at 24 hpt and subjected to immunoblotting with antibodies against GFP, Flag, and β-actin as an internal control. (**B**) Quantification analysis of viral protein expression from (**A**) with Image J, the level of GFP-tagged viral proteins band ratios was normalized to β-actin. (**C**) Western blot analysis of GFP-3D protein levels in BHK-21 cells cotransfected with Flag-HDAC4 or Flag empty vector for 24 h, then cells were treated with MG132 (10 µM), NH_4_Cl (10 mM), Z-VAD-FMK (50 µM), Baf A1 (200 nM), CQ (40 µM), and 3-MA (25 mM) for 12 h, respectively. (**D**) Quantification analysis of viral 3D protein expression from (**C**) with Image J, the level of GFP-3D band ratios was normalized to β-actin. (**E**) Interaction domains identification of cleaved HDAC4 with GFP-3D. Flag-HDAC4 and its cleaved products were cotransfected with GFP-3D for 24 h, then subjected to co-IP analysis with antibodies against GFP, Flag, and β-actin as an internal control. (**F**) Quantification analysis of viral 3D protein expression from (**E**) with Image J, the level of GFP-3D band ratios was normalized to β-actin. (**G**) Colocalization analysis of cleaved HDAC4 products with GFP-3D. BHK-21 cells were cotransfected with indicated plasmids for 24 h and stained with antibodies to Flag (red) and DAPI (blue), and examined under confocal microscopy. The error bars indicated mean ± SD from three independent experiments (**P＜0.01; ***P＜0.001; NS, not significant).

Overexpression of HDAC4 led to substantial degradation of the viral RNA-dependent RNA polymerase 3D protein, whereas other viral proteins were not significantly affected (Fig. 6A and B). This indicated that HDAC4 selectively targets 3D protein for degradation. Treatment with the proteasome inhibitor MG132 restored the levels of 3D, but not treatment with NH_4_Cl, Z-VAD-FMK, Baf A1, CQ, or 3-MA, indicating that 3D is degraded by HDAC4 via the proteasome pathway (Fig. 6C and D). Co-IP verified that the C-terminal fragment of HDAC4 (600–1,084), which contains a deacetylase domain, is responsible for its interaction with the 3D protein (Fig. 6E). Additionally, neither the HDAC4 (1–599) nor the HDAC4 (600–1,084) fragments were able to degrade the 3D protein (Fig. 6E and F). Consistent with the Co-IP results, confocal microscopy revealed that the C-terminal fragment of HDAC4 (600–1,084) colocalized with 3D in the cytoplasm and exhibited a punctate distribution (Fig. 6G). The N-terminal fragment of HDAC4 (1–599), containing the NLS, also colocalized with 3D but in the nucleus (Fig. 6G), it did not interact with 3D in the co-IP assay (Fig. 6E). Collectively, these findings indicate that 3D was degraded in a proteasome-dependent manner and that the cleaved HDAC4 products failed to degrade the viral 3D protein.

### SVV infection induces cytoplasmic redistribution of HDAC4 from the nucleus

**Fig 7:**
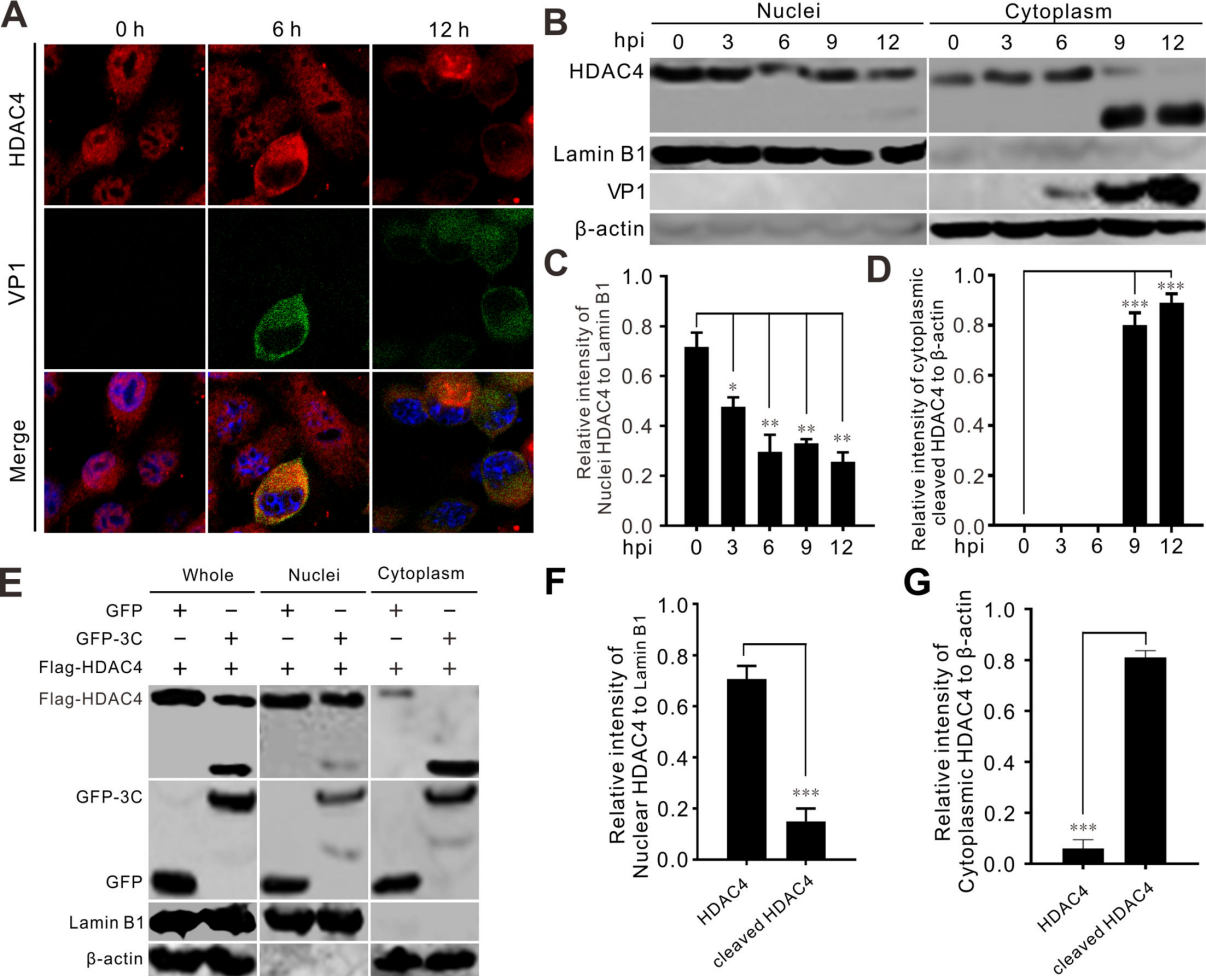
SVV infection induces cytoplasmic redistribution of HDAC4. (**A**) SVV-infected-BHK-21 cells (MOI = 1) were monitored with fluorescence microscopy to analyze the subcellular localization of HDAC4 at 6 and 12 hpi, respectively. Cells were stained with HDAC4 polyclonal antibody (red), viral VP1 monoclonal antibody (green), and DAPI (blue), then examined by confocal microscopy. (**B**) SVV-infected-BHK-21 cells were subjected to nuclear and cytoplasmic fractions at 0, 3, 6, 9, and 12 hpi (MOI = 1). The expression of HDAC4 was determined by immunoblotting. (**C-D**) The graph presents the quantitative analysis of nuclei HDAC4 normalized against Lamin B1 (**C**), and cytoplasmic cleaved HDAC4 normalized against β-actin (**D**), respectively. The error bars represented the mean ± SD from three independent experiments. (**E**) The cytoplasmic and nuclear components were extracted from BHK-21 cells overexpressing Flag-HDAC4 with GFP-3C or GFP empty vector. (**F-G**) The graph presents the quantitative analysis of nuclei HDAC4 normalized against Lamin B1 (**F**), and cytoplasmic cleaved HDAC4 normalized against β-actin (**G**), respectively. The error bars represented the mean ± SD from three independent experiments. (*P＜0.05; **P＜0.01; ***P＜0.001).

Immunofluorescence assays demonstrated that HDAC4 was predominantly located in the nucleus of mock-infected cells (Fig. 7A). However, upon SVV infection, the vast majority of HDAC4 relocated from the nucleus to the cytoplasm (Fig. 7A). Cytoplasmic and nuclear fractionation assays further confirmed that HDAC4 underwent cytoplasmic relocalization in the SVV-infected cells (Fig. 7B through D). These results suggested a constant decline in HDAC4 levels in the nucleus, concomitant with an increase in cleaved HDAC4 in the cytoplasm during late SVV infection (Fig. 7B through D). The cytoplasmic redistribution of HDAC4 was remarkably increased at 9 h post-infection (hpi), along with the expression of VP1 (Fig. 7B through D). HDAC4 was mainly located in the nucleus, whereas following transfection of a GFP-3C expression vector, cleaved HDAC4 translocated from the nucleus to the cytoplasm (Fig. 7E through G). By contrast, transfection of an empty GFP vector did not affect the distribution of HDAC4 (Fig. 7E through G). Collectively, these results indicate that SVV infection induced cytoplasmic redistribution of cleaved HDAC4.

### Cleaved HDAC4 abrogates induction of type I interferon and ISG expression

**Fig 8:**
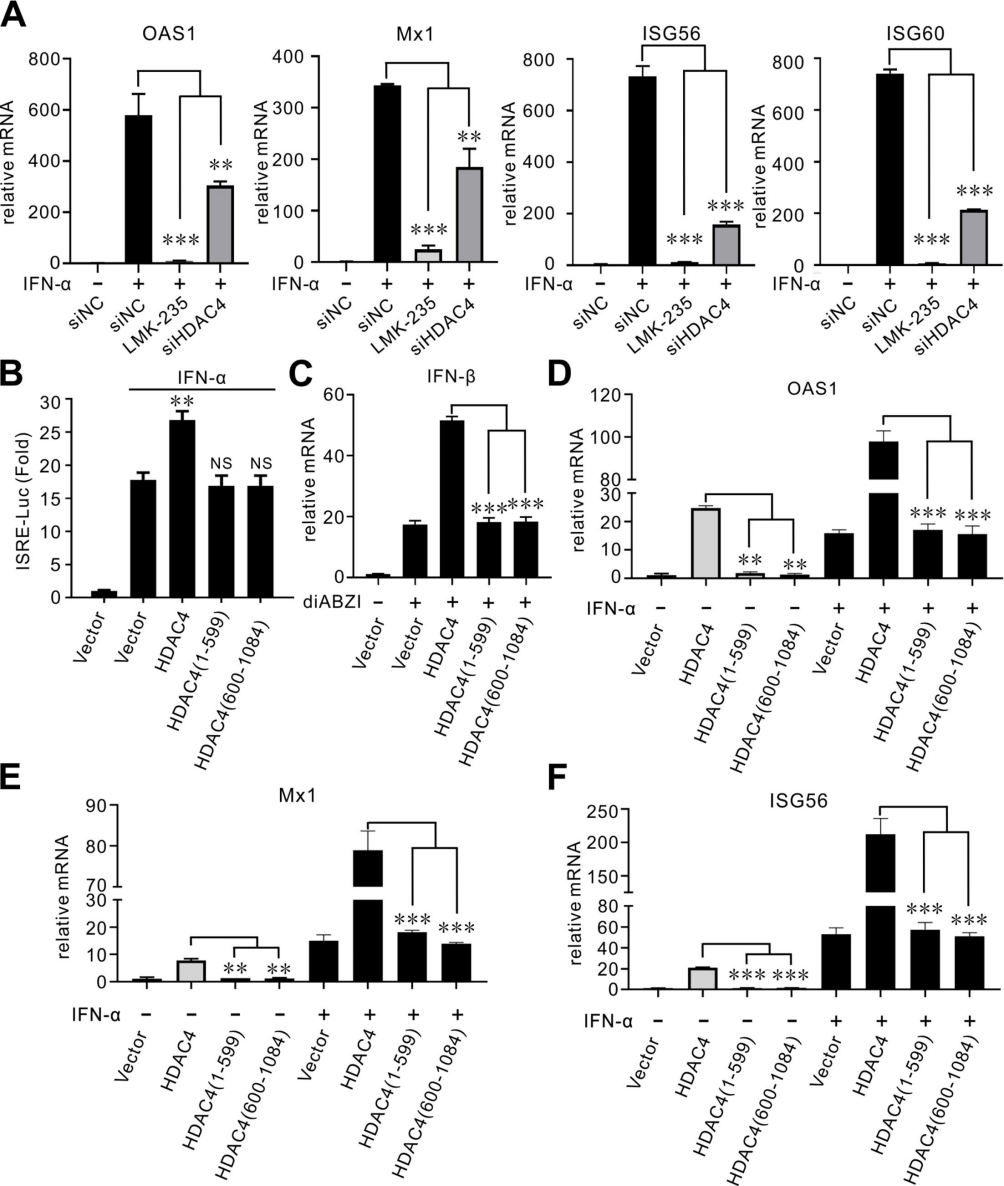
Cleaved HDAC4 abrogates the induction of type I interferon and ISG expression. (**A**) Relative abundance of ISG60, ISG56, OAS, and Mx1 mRNA in LMK-235 treated or siHDAC4-transfected HEK-293T cells after IFN-α (1,000 U/mL) stimulated were determined by qRT-PCR. The level of OAS1, Mx1, ISG56, and ISG60 mRNA was normalized against GAPDH. Data are shown as the mean ± standard deviation (SD) obtained from three independent experiments. (**B**) HEK-293T cells grown in 24-well plates were cotransfected with Flag empty vector, Flag-HDAC4 and its truncation constructs, pISRE-Luc plasmid, and pRL-TK plasmid. At 24 hpt, cell samples were treated with IFN-α (1,000 U/mL) for 12 h, and then luciferase assays were performed. (**C**) HEK-293T cells were transfected with Flag empty vector, Flag-HDAC4, and its truncation constructs for 24, then treated with diABZI for 12 h. The relative abundance of IFN-β mRNA was determined by qRT-PCR. (**D-F**) HEK-293T cells were transfected with Flag empty vector, Flag-HDAC4, and its truncation constructs for 24, then treated with IFN-α (1,000 U/mL) or mock treated with DMEM for 12 h. The relative abundance of OAS1, Mx1, and ISG56 mRNA was determined by RT-qPCR. The error bars represented the mean ± SD from three independent experiments. (**P＜0.01; ***P＜0.001; NS, not significant).

Previous studies have shown that HDAC4 promotes type I IFN signaling. HDAC4 is recruited to IFN-stimulated response element (ISRE) promoters and is required for recruitment of STAT2 (24). We treated cells with LMK-235, an inhibitor of class II HDACs, and showed that this treatment blocked promotion of mRNA expression of 2′−5′-Oligoadenylate synthetase 1 (OAS1), Mx1, ISG56, and ISG60 (Fig. 8A). Similar results were obtained following siRNA-mediated knockdown of HDAC4 (Fig. 8A). Given that HDAC4 is cleaved into two fragments by SVV 3C^pro^, we wondered whether cleaved HDAC4 retained its capacity to trigger ISRE promoter activation and subsequent ISG induction. To address this question, HEK-293T cells were cotransfected with an ISRE-Luc reporter plasmid, and pRL-TK, Flag-HDAC4, Flag-HDAC4 (1–599), Flag-HDAC4 (600–1,084), or a Flag empty vector for 20 h, and then the cells were treated with IFN-α for 10 h. The results of the luciferase activity assays indicated that expression of HDAC4-WT markedly boosted ISRE promoter activity, whereas the cleavage products did not (Fig. 8B). HDAC4 significantly augmented diABZI-induced mRNA expression of IFN-β (Fig. 8C). HDAC4 notably enhanced mRNA expression of OAS1, Mx1, and ISG56 (Fig. 8D through F). However, induction of these ISGs disappeared in cells expressing either of the two cleaved HDAC4 products, HDAC4 (1–599) or HDAC4 (600–1,084) (Fig. 8D through F). HDAC4 also significantly augmented IFN-α-induced mRNA expression of OAS1, Mx1, and ISG56 (Fig. 8D through F). By contrast, cleaved HDAC4 products lose the capacity to activate the type I interferon pathway (Fig. 8D through F). Taken together, these results showed that HDAC4 is essential for type I IFN signal transduction and that cleavage by SVV 3C^pro^ abrogated its ability to induce ISG expression.

## DISCUSSION

Most proteins undergo acetylation following translation, and HDACs are essential core enzymes regulating acetylation that play a significant role in various processes, including in viral infections (18–24). Accumulating evidence has revealed that HDACs are critical regulators of the IFN-I pathway and antiviral immunity (18–24). In turn, viruses have established finely tuned mechanisms that target HDACs to promote efficient viral replication. In the present study, we found that SVV infection reduced the levels of HDAC4 by inducing cleavage of HDAC4. The viral 3C^pro^ was identified as the enzyme responsible for this cleavage. HDAC4 acts as an intrinsic cellular restriction molecule against SVV infection by selectively targeting the viral RNA-dependent RNA polymerase 3D for proteasomal degradation. SVV 3C^pro^-mediated cleavage of HDAC4 completely abolished its antiviral effects, as evidenced by the fact that the cleaved HDAC4 fragments were unable to activate IFN signaling and ISG expression.

Recently, two swine enteropathogenic coronaviruses, PEDV and PDCoV, were shown to induce the degradation of several HDACs, including HDAC1, HDAC2, and HDAC6 (20–22). PEDV infection downregulated HDAC1 expression, leading to decreased expression of ISGs, and the PEDV N protein was shown to be responsible for downregulating HDAC1 expression (22). PDCoV infection suppressed cellular deacetylase activity and reduced the expression of some HDACs (20, 21). In the present study, we found that HDAC4 was degraded and cleaved in SVV-infected BHK-21, HEK-293T, and PK-15 cells (Fig. 1A through F). However, unlike PDCoV, SVV infection did not affect HDAC4 transcription (Fig. 1G through I). Ectopic expression of HDAC4 robustly inhibited SVV replication (Fig. 2E, F, H and J). Conversely, siRNA-mediated knockdown of HDAC4 significantly enhanced SVV replication (Fig. 2C, D, G, and I). This indicated that HDAC4 is an antiviral factor against SVV infection.

HDAC4 is composed of two domains: the C-terminal domain, which is highly conserved and has deacetylation activity, and the N-terminal domain, which mediates protein targeting and has a vital role in the function of HDAC4. Through deacetylation, HDAC4 promotes the formation of a tight histone-DNA complex, which, in turn, suppresses both gene replication and transcription (30). Through protein targeting, HDAC4 regulates diverse signaling pathways (30) and regulates a wide range of cellular functions, including gene transcription and cell growth and development (31). We found that SVV 3C^pro^ was responsible for the cleavage and degradation of HDAC4, and the two specific HDAC4 cleavage bands were not present in cells transfected with the other viral proteins (Fig. 3A). Lopinavir is a protease inhibitor that has been employed in the clinical treatment of human immunodeficiency virus (HIV). Enterovirus D68 (EV-D68) 3C^pro^ cleaves TAR DNA-binding protein 43 kDa (TDP-43), and Lopinavir directly inhibits 3C-mediated TDP-43 cleavage by inhibiting the enzymatic activity of 3C^pro^. Moreover, Z-VAD-FMK exerts no suppressive effect on EV-D68 3C^pro^-mediated or EV-D68 infection-mediated TDP-43 cleavage (32). Methylated forms of the Z-VAD-FMK show a significant inhibitory effect on picornaviral 2A^pro^ in eIF4GI cleavage, it is not clear whether Z-VAD-FMK blocks only 2A^pro^ or whether both 2A^pro^ and 3C^pro^ are inhibited (33). The cleavage of HDAC4 induced by SVV 3C^pro^ was suppressed following the treatment with Lopinavir in a dose-dependent manner (Fig. 3C and D), Z-VAD-FMK has no inhibitory impact on the cleavage of HDAC4 mediated by SVV 3C^pro^ or SVV infection (Fig. 3E through H). These results indicated that Z-VAD-FMK does not inhibit 3C^pro^ activity. Interestingly, we also found that 3C^pro^ proteins from three other viruses (CVB3, HRV, and EV71) also cleaved HDAC4. However, the cleaved bands were smaller than those induced by SVV infection (Fig. 3I), indicating that the specificity of HDAC4 cleavage by SVV 3C^pro^ differs from that of other picornaviruses. HDAC4 restricted vaccinia virus (VACV) replication, and viral C6 protein targeted HDAC4 for proteasomal degradation (24). HDAC4 also possesses anti-IAV properties, and the viral RNA endonuclease PA-X downregulates HDAC4 (18). These results indicate that different viruses have evolved the same strategies to antagonize host antiviral factors. We found that 3C^pro^ cleaved HDAC4 at Q599, separating the N- and C-terminal (deacetylase) domains (Fig. 4A). The cleaved HDAC4 products did not inhibit SVV replication (Fig. 4D through F). However, a more potent inhibitory effect was observed in cells transfected with the mutant HDAC4-Q599A (Fig. 4D through F). These findings indicate cleavage of HDAC4 completely abrogates its ability to restrain SVV replication and that mutation of the 3C^pro^ cleavage site in HDAC4 augments its antiviral effect.

HDAC6 suppressed viral replication of PDCoV by degrading the viral nsp8 protein through deacetylation and targeting it for ubiquitination (21). Five SVV proteins, Lpro, VP3, VP1, 3C, and 3D could colocalize with HDAC4 and exhibited punctate distribution patterns (Fig. 5A). Two of these, VP1 and 3C, induced translocation of HDAC4 from the nucleus to the cytoplasm and produced cytoplasmic puncta (Fig. 5A). The interactions between HDAC4 and Lpro, VP3, VP1, 3C, and 3D were verified using Co-IP (Fig. 5B). HDAC4 colocalized and interacted with five structurally very different viral proteins (Fig. 5A and B), indicating that HDAC4 is involved in other biological function during SVV infection. The C-terminal deacetylase domain of HDAC4 was responsible for its interaction with SVV 3D (Fig. 5C and D). HDAC4 also targeted SVV 3D protein for degradation via the proteasomal pathway (Fig. 6A through D) and cleavage of HDAC4 by 3C abrogated its ability to degrade 3D (Fig. 6E and F). Thus, we speculated that cleavage of HDAC4 abrogated its ability to inhibit SVV replication owing to HDAC4-induced degradation of 3D. In contrast to class I HDACs, which reside in the nucleus and deacetylate histones, class IIa HDACs move between the nucleus and cytoplasm because of reversible phosphorylation of the N-terminal domain. Unphosphorylated class IIa HDACs in the nucleus associate with chromatin and suppress transcription. When phosphorylated, these HDACs leave the nucleus, thus freeing their target genes from transcription repression (15, 34). SVV infection induced cytoplasmic relocalization of HDAC4; after SVV infection, HDAC4 was not present in the nucleus, but was found in the cytoplasm (Fig. 7A). Cytoplasmic and nuclear fractionation showed that cytoplasmic HDAC4 was mainly the cleaved form and that 3C^pro^ was responsible for its translocation (Fig. 7B through G).

Type I IFN plays a crucial role in the host’s defense against viruses. Its antiviral activity is largely due to the induction of JAK-STAT signaling and ISG expression. Several studies have shown that HDACs are crucial regulators of the type I interferon (IFN) pathway. In the context of viral infection, HDAC1 plays a crucial role in enabling transcriptional activation of ISGs through IRF3 (35–37). HDAC1 co-precipitated with STAT1 and STAT2 (23). HDAC6 (38, 39), HDAC2 (36), and HDAC9 (40) promoted the expression of type I IFN. HDAC also modulated the expression of ISGs, as reflected by reduced ISG transcription in cells treated with the HDAC inhibitor TSA (35) and in cells lacking HDAC1 (23, 41), HDAC2 (41), or HDAC3 (42). HDAC4 coprecipitated with STAT2 and was recruited to IFN-stimulated response element (ISRE)-containing promoters to enhance type I IFN signaling (24). In our study, inhibition of HDAC4 expression by the HDAC inhibitor LMK-235 or siRNA-mediated knockdown significantly impaired IFN-α-stimulated transcription of ISGs (Fig. 8A). Conversely, overexpression of HDAC4-WT induced IFN-β and ISG expression, whereas expression of the cleaved HDAC4 product did not induce ISGs (Fig. 8B through F). The results showed that HDAC4-WT, but not the two cleaved fragments of HDAC4 (1–599) and (600–1,084), increased IFN-α-induced ISG expression (Fig. 8D through F). This showed that both domains of HDAC4 are essential for activating type I IFN signaling. Inhibition of HDAC4 increased SVV replication, likely due to decreased expression of IFN-β and ISGs. These results indicate that the function of HDAC4 during SVV infection is related to the expression of ISGs. HDAC4 also participates in the innate antiviral response to IAV (18), and in HDAC4-depleted cells, there was an obvious reduction in p-STAT1 levels and ISGs, such as IFITM3, ISG15, and viperin (18).

**Fig 9:**
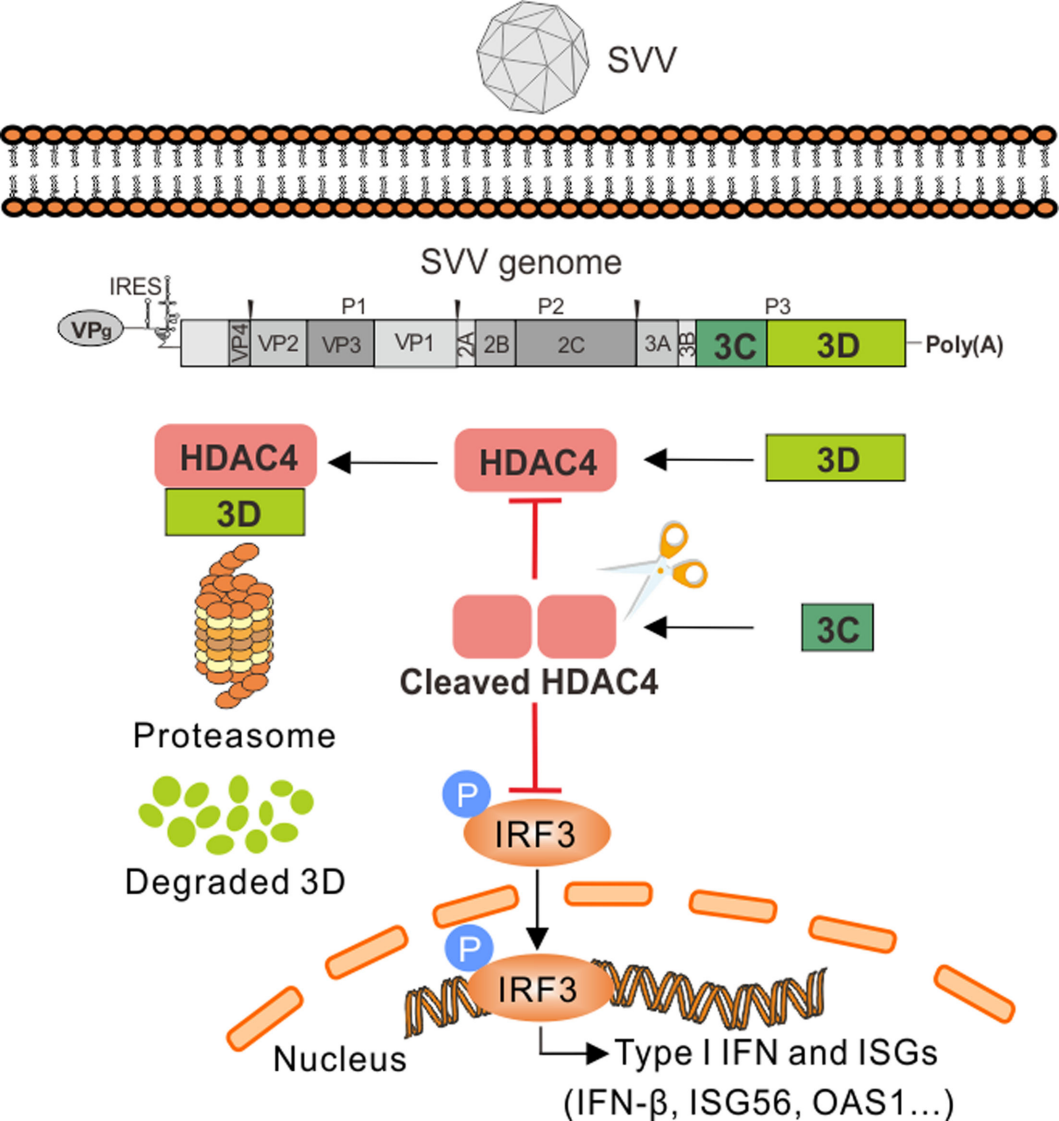
A proposed model for SVV antagonizes type I IFN signaling by targeting HDAC4. HDAC4 enhances type I interferon signaling activation and limits SVV replication by targeting viral RNA-dependent RNA polymerase 3D protein for degradation. SVV 3C^pro^ induces degradation and cleavage of HDAC4, resulting in the cleaved HDAC4 products abrogating its ability to restrict viral replication and activate the type I IFN signaling pathway.

In summary, our study provides evidence that HDAC4 is a host restriction factor for SVV replication that functions by targeting the viral 3D protein for degradation and that HDAC4 is degraded and cleaved during SVV infection (Fig. 9). Cleavage of HDAC4 by SVV 3C^pro^ abrogated its ability to limit viral replication. In addition, the cleaved HDAC4 products cannot activate type I IFN. Our findings reveal a novel mechanism of SVV evasion of the host innate immunity responses by targeting HDAC4.

## Data Availability

The data that support the findings of this study are available from the corresponding author upon reasonable request.
